# Many Faces of Regulatory T Cells: Heterogeneity or Plasticity?

**DOI:** 10.3390/cells13110959

**Published:** 2024-06-01

**Authors:** Varvara G. Blinova, Dmitry D. Zhdanov

**Affiliations:** 1 Laboratory of Medical Biotechnology, Institute of Biomedical Chemistry, Pogodinskaya st. 10/8, 119121 Moscow, Russia; v.02.blinova@gmail.com; 2 Department of Biochemistry, People’s Friendship University of Russia Named after Patrice Lumumba (RUDN University), Miklukho-Maklaya st. 6, 117198 Moscow, Russia

**Keywords:** regulatory T cells, heterogeneity, plasticity, phenotype switch, treg-based therapy

## Abstract

Regulatory T cells (Tregs) are essential for maintaining the immune balance in normal and pathological conditions. In autoimmune diseases and transplantation, they restrain the loss of self-tolerance and promote engraftment, whereas in cancer, an increase in Treg numbers is mostly associated with tumor growth and poor prognosis. Numerous markers and their combinations have been used to identify Treg subsets, demonstrating the phenotypic diversity of Tregs. The complexity of Treg identification can be hampered by the unstable expression of some markers, the decrease in the expression of a specific marker over time or the emergence of a new marker. It remains unclear whether such phenotypic shifts are due to new conditions or whether the observed changes are due to initially different populations. In the first case, cellular plasticity is observed, whereas in the second, cellular heterogeneity is observed. The difference between these terms in relation to Tregs is rather blurred. Considering the promising perspectives of Tregs in regenerative cell-based therapy, the existing confusing data on Treg phenotypes require further investigation and analysis. In our review, we introduce criteria that allow us to distinguish between the heterogeneity and plasticity of Tregs normally and pathologically, taking a closer look at their diversity and drawing the line between two terms.

## 1. Introduction

The subset of regulatory T cells (Tregs) is a critical player in the regulation of immune homeostasis in normal and various pathological conditions, such as cancer, autoimmune diseases or organ transplantation. Tregs support an easily perturbed mechanism that maintains the balance between immune response and self-tolerance, and they exert their functions in a variety of cell contact-dependent and contact-independent ways. Numerous studies have reported an obvious need for the presence of Tregs, e.g., to prevent the development of spontaneous autoimmunity or graft-versus-host disease (GvHD), and in cancer development, these cells facilitate tumor immune evasion [1]. A number of clinical trials are being conducted to explore the possibility of using Tregs as an adoptive cell therapy (ACT) [2,3]. To date, several results from clinical trials of ACT using ex vivo expanded autologous Tregs have demonstrated the safety and promising efficacy of Tregs applications [4,5,6,7,8]. However, the practical implementation of ACT approaches with Tregs is currently impossible due to several limitations. These limitations can be divided into the following categories: biological, technical and economic. Avoiding economic, biological and technical limitations is particularly highlighted in our work. Technically, expansion strategies that allow obtaining sufficient numbers, purity and sterility of Treg cell products still need to be developed [9]. On the biological side, the development of several pathologies may be associated with dysfunctional Tregs [9], and the detailed study of factors that define their proper functionality is under major consideration. The selection of Tregs for clinical trials is complicated by the existence of conflicting information on the role of different Treg subpopulations in the development of certain pathological conditions. Existing contradictions may be partly explained by the choice of different phenotypes to identify Tregs. We can only distinguish between different subpopulations by the markers they express. Initially, Tregs were characterized as CD3^+^CD4^+^CD25^hi^ cells, but CD25 is also expressed by conventional T cells. Many markers that researchers have used to distinguish and identify Tregs (cytotoxic T lymphocyte antigen 4 (CTLA-4), glucocorticoid-induced tumor necrosis factor receptor (TNFR)-related protein (GITR), inducible T-cell costimulator (ICOS), Helios) were described later, but, unfortunately, they are not exclusively expressed by Tregs. Of these, the transcription factor Forkhead box P3 (FoxP3) is considered to be the most specific and plays a major role in the differentiation, maturation and acquisition of suppressive function by Tregs. A negative correlation between CD127 and FoxP3 expression was also found, characterizing Tregs as cells with low CD127 expression [10]. Of note, low-to-absent CD127 expression is observed in steady-state Tregs, whereas during activation, they significantly upregulate these molecules [11]. Moreover, CD127 in combination with other markers can be employed for Treg identification with greater accuracy in healthy individuals. In pathology, the pool of CD4^+^CD25^+^CD127^-^ cells can be contaminated with FoxP3^-^ cells, which necessitates a more precise analysis and separation of non-regulatory cells from regulatory ones [12,13]. In this regard, to identify steady-state Tregs in healthy individuals, the CD3^+^CD4^+^CD25^high^CD127^low^FoxP3^+^ phenotype is recommended, and in pathology, the CD3^+^CD4^+^CD25^high^FoxP3^+^ phenotype can be used. A large number of markers expressed in addition to FoxP3 indicate the development of distinct Treg subpopulations. It is not entirely clear whether these subpopulations exist independently and develop from distinct progenitors or whether the appearance of a marker is a transient response to changing microenvironmental cues. If the former is the case, we can refer to cellular heterogeneity driven by changes in the genome and transcriptome, resulting in the emergence of distinct cell progenitors [14,15]. The second case is intrinsically related to cellular plasticity, which defines the ability of cells to change their phenotype in response to external stimuli without genetic mutations [16].

Considering the above, the choice of a marker to identify the Treg subpopulation that represents its state and origin is of primary importance. The use of different Treg phenotypes in studies in the presence of conflicting data requires a closer look at their biology and functions.

The aim of this review is to clarify the data on the generation, differentiation and maturation of different Treg subpopulations, to compare their functional activity depending on the presence of certain markers, and to study the role of different subpopulations in the development of pathological processes, thereby shedding light on their potential use as adoptive Treg cell-based therapy.

## 2. Heterogeneity and Plasticity of Cells

### 2.1. Cellular Heterogeneity and Plasticity of the Cell in General

The distinction between cellular heterogeneity and plasticity raises some issues due to the interchangeable use of the two terms. The heterogeneity and plasticity of cells under various conditions, e.g., during homeostasis, in cancer, in autoimmune diseases and in infectious diseases, are constantly discussed, and their exact definitions may vary according to different authors. However, stem cells seem to be one of the most studied subjects in which the definitions of the two terms are unambiguous. In the case of stem cells, heterogeneity is described as moderate changes in the transcriptome and function that allow adaptation to the microenvironment, organ or anatomical location [17]. Different aspects of cellular physiology, such as epigenetics, transcription, mitosis, signal transduction and metabolic regulation, vary among heterogeneous stem cells [18]. For example, epigenetic states, particularly variations in chromatin accessibility, nucleosome positioning, histone modifications and DNA methylation at enhancers and promoters, positively correlate with gene expression profiles in established cell lines [19]. Importantly, these adaptations do not result in the loss or acquisition of classical cellular identity markers, which is distinct from cellular plasticity. Plasticity is therefore defined as the adaptation of a cell to its environment, resulting in a complete change in cell identity. This is associated with the loss or acquisition of classical cell identity markers and, when applied to stem cells, includes the so-called trans-differentiation and reversal of this process [17]. Stem cell plasticity occurs during the process of differentiation and self-renewal induced by aging or pathological conditions. At this time, cells can switch their functions, phenotype and/or composition [18]. Regardless of stem cells, cellular plasticity is observed and is being intensively studied in tumors, where phenotype switching is a key contributor to therapy evasion [16]. A good example is the epithelial-to-mesenchymal transition (EMT) in response to microenvironmental stress. This process is represented by the loss of apical–basal polarity and epithelial cell junctions, accompanied by the acquisition of mesenchymal properties. Because EMT is completely reversible, it demonstrates cellular plasticity in cancer in the absence of genetic mutations [16]. Thus, heterogeneity and plasticity are two intrinsic properties of different cellular populations that allow either stable genetic-driven or reversible genetic-independent adaptations to novel normal or pathological conditions. In our review, we introduce criteria that help distinguish between the two phenomena by analyzing the available data (Table 1). Factors and mechanisms explaining their occurrence are not fully understood and require further investigation.

**Table 1:** Criteria for determining cellular heterogeneity and plasticity.

Heterogeneity	Plasticity	Reference
Appears due to changes in genome and transcriptome.	No genetic alterations occur.	[16,17,20]
Once initiated during the developmental process, becomes fixed and remains stable under physiological conditions.	Unstable and reversible.	[18]
Classical cellular identity markers remain during adaptation.	Loss or acquisition of cellular identity markers.	[17]
Different fates of heterogenous subpopulations	Due to reversibility, fates may not differ.	[18]
Regulation by different signaling pathways.	Signaling pathways change in response to environmental cues.	[18]
Different functional steady-state characteristics: self-renewal (for stem cells), proliferation, differentiation and lifespan.	Plasticity results in altered functioning.	[21]

However, these criteria are not strict and may in some cases characterize both plasticity and heterogeneity. The main discrepancy arises from the genetic nature of heterogeneity.

### 2.2. Cellular Heterogeneity and Plasticity among Tregs

Focusing on Tregs, this cellular subset is no exception in terms of both heterogeneity and plasticity. Evidence for these two cellular properties can be found starting from the development of Tregs in the thymus and can be observed later during maturation and function and in association with pathology. A major obstacle to the identification and comparison of functional properties and consideration for therapeutic purposes is the plasticity of Tregs, which exhibit phenotype switching. By acquiring novel markers, Tregs show different functional properties that may be associated with acquired pro-inflammatory activity [22]. Furthermore, cellular plasticity is the major contributor to Treg instability. The reasons leading to heterogeneity and plasticity are poorly understood, but factors that define the dichotomy in thymic Treg development and thus the appearance of two heterogeneous subpopulations are actively discussed [23]. Similar to cellular heterogeneity and plasticity in general, heterogeneity in Tregs represents a genetically determined irreversible property, whereas plasticity is a reversible adaptation that exhibits unstable phenotype switching. In Tregs, other factors such as TCR density and FoxP3 expression influence the presence of two phenomena that we will discuss in detail. Below in Table 2, we note the criteria that help navigate Treg heterogeneity and plasticity. These criteria are related to the criteria for determining cellular heterogeneity and plasticity presented in Table 1. The following sections explore the aspects mentioned here.

**Table 2:** Criteria for determining Treg heterogeneity and plasticity.

Heterogeneity	Plasticity
Initially, new different subpopulations arise due to different developmental programs in the thymus.	New subpopulations emerge as a response to changing environments (inflammation, tumor microenvironment).
Different cell precursors.	A common cell precursor.
Expresses TCRs with different affinities for self-antigens.	TCR-independent.
Exhibits differences in the composition of its transcriptome and relies on the use of different genetic enhancer elements.	Lineage commitment is dependent on epigenetic program, transcription factors and expression of FoxP3.
Irreversible development of subpopulation.	Development may be reversible.
Temporary existence of subpopulation with stable expressed markers.	Instability of expressed markers. Ability to reversibly lose and acquire markers.
Stability of functional properties (mechanisms and released cytokines) in one subpopulation, diverse functional capacities between heterogenous subpopulations.	Functional activity may change under certain conditions.

## 3. Markers Suitable for Determination of Tregs

As mentioned above, several markers have been described to identify Tregs in normal and pathological conditions. Some of them are stable, whereas others appear in response to a new environment and reflect changes in the Treg state. Currently, the most accepted marker of the Treg lineage is FoxP3. FoxP3 is a transcription factor that is critical for the differentiation and maturation of Tregs and for the acquisition of their suppressive function. Mutations in the FoxP3 gene cause highly lethal immunodysregulation, polyendocrinopathy, enteropathy and X-linked (IPEX) syndrome [24]. The main event in the pathogenesis of IPEX is Treg dysfunction leading to multi-organ autoimmunity [25]. Interestingly, the clinical manifestation of IPEX, as well as the Treg phenotype, depends on the impaired expression of a specific FoxP3 exon as a result of a mutation [26]. Alternative splicing of FoxP3 results in the existence of four isoforms of the FoxP3 protein: a full-length variant (FL), variants with deletions of either exon 2 (∆2 variant) or 7 (∆7 variant) and a deletion of both exons (∆2∆7 variant) [27]. It is noteworthy that there are differences in the numbering of these exons in the literature, and exons 2 and 7 (protein coding) are referred to as 3 and 8, respectively, in some sources, considering the non-coding exon as the first [28]. Importantly, Tregs expressing the FL FoxP3 variant show higher suppressive activity and proliferation rates compared to those expressing truncated FoxP3 variants [27]. Impaired FoxP3 isoform ratios are observed in several diseases, affecting the Treg immunophenotype. In a recent paper, we demonstrated that patients suffering from amyotrophic lateral sclerosis have reduced expression of FL FoxP3, and the expression of other truncated variants predominates [29]. The induction of selective FL FoxP3 expression resulted in the appearance of a more suppressive and proliferative phenotype [29]. Therefore, FoxP3-detecting antibodies must be used with caution when identifying Tregs. Commercially available antibody clones react with different FoxP3 epitopes. For example, the binding of the PCH101 antibody to the epitope located at the amino terminus of human FoxP3 allows the detection of all FoxP3 isoforms, whereas the 150D antibody recognizes only the epitope encoded by exon 2 [30].

The majority of mouse and human Tregs express the zinc finger transcription factor Helios [31], which should also be considered when selecting markers to identify Tregs. Previous studies have not been able to fully elucidate the exact role of Helios in human Treg stability and function. However, one study demonstrated the role of Helios in cellular activation and division [32]. Intriguingly, a recent paper showed that Helios ablation does not negatively affect the natural or memory Treg phenotype, function or stability in inflammation, but it would be useful to include Helios in addition to FoxP3 as a marker during Treg expansion to monitor their stability and purity in the context of Treg-based therapy [33]. In light of the preceding study, it seems Helios may also serve as a marker of activation and proliferation; however, future studies are needed.

Other surface and intracellular Treg markers, listed below in Table 3, are either also used for the identification and phenotypic characterization of Tregs or may reflect their maturation or functional state. Our review is focused on human Tregs, although it is also necessary to consider changes in Treg markers that have been detected in mice. Although mice and humans have similar Treg transcription profiles, markers predominantly detected in mice should be properly validated in humans, and such markers are marked in the table. Markers not included in the table are being studied in more detail, either in relation to a specific pathology or to a specific aspect of Treg biology, and are discussed in the appropriate sections.

**Table 3:** Treg cell markers suitable for determination of Treg subpopulation.

Marker	Biological Functions	Association with Tregs	References
CD25	The α chain of IL-2 receptor. Has an impact on T cell proliferation, activation-induced cell death, the actions of Tregs and effector (Teff) T cells [34].	High expression of CD25 remains one of the predominant characteristics of conventional Tregs. The subset of FoxP3^+^ Tregs with the highest levels of CD25 also has the phenotype with the strongest immune-suppressive activity.	[35]
CD127	The α chain of IL-7 receptor. Dynamic regulation of the T cell compartment, effects on lymphocyte differentiation and cellular immunity [36].	Low expression of CD127 remains to be one of the predominant characteristics of conventional Tregs. Expression is significantly upregulated during activation, whereas at the steady state, Tregs express low-to-absent levels of CD127. Differential CD127 expression on Treg depends on their localization and activation status.	[11]
CD39	Ectoenzyme (ecto-nucleotide triphosphate diphosphohydrolase 1). Strategic role in calibrating the duration, magnitude and chemical nature of purinergic signals delivered to immune cells through the conversion of ADP/ATP to AMP and AMP to adenosine; attenuating immune responses in cancer [37,38].	Expression of CD39 on Tregs shows high interindividual variation and is especially high at sites of inflammation. CD39^+^ Tregs are thought to be highly active, suppressive and capable of secreting IL-10. CD39 expression is increased in CD25^low^ Tregs but reduced in CD25^high^ Tregs. Human CD39^high^ Tregs sustain higher FoxP3 expression levels and stronger suppressive abilities even in the presence of inflammatory cytokines in vitro.	[39,40]
CTLA-4	Inhibitory receptor belonging to the CD28 immunoglobulin superfamily. Regulates the costimulation of T cells and is required for Treg cell functions. Has an impact on thymic development, T cell priming, peripheral tolerance [41,42].	Constantly expressed. Highly expressed during immunosuppressive activity. CTLA-4 defines Tregs with strong suppressive activity.	[43]
GITR	Member of the TNF receptor superfamily. Regulates immune responses by providing costimulatory signals that enhance responder T cell functions, including activation, differentiation, survival and memory formation; macrophage polarization [44].	Constantly expressed. Role is controversial: causes instability, Treg cell depletion and decreases Treg suppressive function while inducing Treg proliferation and expansion in vitro.	[45,46]
TIGIT	T cell immunoreceptor with immunoglobulin and ITIM domain. Transmembrane glycoprotein receptor. Downregulates T and NK cell functions, and is a key inhibitor of anti-tumor responses [47].	Highly expressed by Tregs following their activation. Increased suppression by TIGIT^+^ Tregs compared to TIGIT^−^ Treg cells. TIGIT^+^ Treg cells specifically inhibit proinflammatory T helper (Th)1 and Th17 cells but not Th2 cell responses	[48,49]
CD120b (TNFR2)	Tumor necrosis factor receptor 2. Anti-inflammatory activities, FR protective effects on oligodendrocytes, cardiomyocytes and keratinocytes, fulfills immunosuppressive activities by its effects on different types of immune cells [50].	Highly and continuously expressed by activated Tregs Interaction between TNF-α and TNFR2 critically affects the activation, expansion and phenotypic stability of Tregs	[51,52]
LAP	Latency-associated peptide. The amino-terminal domain of the transforming growth factor-β (TGF-β) precursor peptide forms a latent TGF-β complex. Modulates monocyte recruitment [53].	LAP^+^ Tregs are highly demethylated in Treg-specific demethylation region (TSDR) and suppressive in vitro and in vivo.	[54]
CCR6	C-C chemokine receptor protein. Has a key role in orchestrating the migration of immune cells to inflammatory sites, maintaining immune homeostasis and the balance of immune system integrity [55].	High proportions of CCR6^+^ Tregs have been observed in various inflammatory diseases.	[56]
CD223 (LAG-3)	Lymphocyte-activation gene-3. An immune checkpoint receptor protein. Inhibitor of cell proliferation, immune function, cytokine secretion, and homeostasis [57].	Constantly expressed. LAG-3 on Tregs limits proliferation and functionality by repressing pathways that promote the maintenance of Tregs at inflammatory sites.	[58,59]
GARP (LRRC32)	Glycoprotein A repetitions predominant. A membrane receptor for latent TGF-β. Regulates the availability of membrane-bound latent TGF-β and modulates its activation on Tregs and platelets [60].	GARP is expressed on the surface of activated Tregs and is a specific marker for human Tregs having high suppressive activity.	[61]
GPA33	Glycoprotein A33. A cell surface antigen. Associated with immune dysregulation [62].	High expression of GPA33 defines CD4^+^CD25^+^CD127^–^FoxP3^+^Helios^+^ Tregs that are unable to produce conventional T (Tconv) cells effector cytokines.	[63]
CD137 + CD154−	CD137 is a costimulatory receptor and a member of the TNFR family. Costimulator for T cell activation. Promotes natural killer (NK)-cell proliferation and cytokine secretion [64].	Marker of antigen-activated FoxP3+ Tregs CD137^+^CD154^−^ Tregs are highly efficient at inhibiting effector T (Teff) cell proliferation. Concrete role depends on the type of immune response.	[65]
Nrp-1	Neuropilin-1. A transmembrane glycoprotein. Involved in axon guidance and angiogenesis [66].	Enriched in mouse Tregs and poorly expressed in humans. Expression of Nrp-1 on the surface of induced Tregs (iTregs) enables the identification and isolation of an iTreg subset that has superior suppressive function under inflammatory conditions. Nrp-1 is selectively higher expressed in peripheral lymphoid organs than in thymic Tregs.	[67,68]
FR4	Folate receptor 4. Isoform of folic acid binding protein. Expressed in mice. Highly homologous to human FR-δ. Has been hypothesized to play a potential role in immune responses [69].	FR4 overexpression enhances Treg proliferation in vitro. Expressed in natural and peripheral Tregs.	[69]
CD103	Integrin αE. Mediates cell adhesion, migration and lymphocyte homing of cells through interaction with E-cadherin [70].	CD103^+^ Tregs show an increased capacity to accumulate in inflammatory sites. CD103 is involved in in situ Treg function within the skin and is associated with higher expression of FoxP3.	[71,72]
CD44	Cellular receptor for hyaluronic acid. Involved in various cellular processes such as motility, survival and adhesion.	Ligand of CD44. High-molecular-weight hyaluronan (HMW-HA) promotes Treg-mediated suppression.	[73]
CD45RA/RO	Receptor protein tyrosine phosphatase. Plays a role in initiation of T cell receptor signaling [74].	Marker of differentiational state of Tregs.	[75]
CD62L	The leucocyte adhesion molecule L-selectin is a transmembrane lectin receptor. Regulates entry of naïve and central memory T cells into lymph nodes and activated CD8+ T cells to sites of virus infection [76].	Enables Treg homing into secondary lymphoid organs.	[77]
CCR7	CC-chemokine receptor 7. Involved in localizing dendritic cells and T cells to lymph nodes. Implicated in peripheral tolerance induction [78].	Enables Treg homing into secondary lymphoid organs.	[77]
CD31 (PECAM-1)	Highly glycosylated Ig-like membrane receptor. Has adhesive and signaling functions in both the immune and vascular systems [79].	Marker of Treg differentiation.	[80]
ICOS	A homodimeric protein. The third member of the CD28 super family.	Involved in the production, proliferation and survival of Tregs.	[81]
FoxP3	Transcriptional factor. Acts to promote immune tolerance [82]	Expression of FoxP3 remains to be one of the predominant characteristics of conventional Tregs. Transcriptional factor, plays a master role in viability and suppressive activity of Tregs. Expression is discontinuous and depends on TSDR methylation status.	[83]
Gal-1	Galectin-1. A glycan-binding protein. Involved in cell growth and migration, inflammation, angiogenesis and promoting nervous system development, muscle differentiation, tumor progression, mediating evasion of cancer immune surveillance, immune tolerance in early pregnancy and cell adhesion.	The effects of galectin-1 on Treg cells are context-dependent. Blockade of galectin-1 markedly impairs the inhibitory effects of Tregs.	[84,85]
Helios	A member of the Ikaros gene transcription factor family [86].	High Helios expression marks stable Tregs, whereas Helios^mid^ identifies destabilized Tregs, but Helios expression per se does not have a direct role in maintaining this lineage-committed state. Helios ablation does not negatively affect the natural Treg or memory Treg phenotype, function or stability in inflammation. Helios identifies but is not required for stable human Tregs.	[33]
PD-1 (CD279)	Programmed cell death-1. Type I transmembrane protein belonging to the immunoglobulin superfamily [87].	PD-1 modulates Treg generation and its immunosuppressive properties.	[87]

## 4. Markers Associated with Differentiation and Maturation of Tregs

Cell differentiation is a complex process by which cells acquire their phenotypic or functional type. The differentiation of Tregs is based on the development of this cell lineage from its thymic and peripheral precursors and, as has been proposed, can be divided into several trajectories [23]. These trajectories depend on the location, genetic and epigenetic programs and the presence of inflammatory or other pathological conditions. Depending on the cumulative effect of these factors during differentiation, differences in the phenotypic characteristics of Tregs develop. These differences are caused by, result from or are accompanied by the expression of various markers. The differentiation process can take place in the thymus and extrathymically in the periphery with the further development of thymic (tTregs) or peripheral (pTregs) Tregs.

### 4.1. Thymic Development

Numerous attempts have been made to identify markers that define tTregs. Nrp-1 and Helios were considered to be the most promising candidates for the identification of thymus-derived Tregs. However, they were later found to be expressed not only by tTregs but also in the periphery under conditions that are not fully understood [88]. Helios is induced during T cell activation and proliferation and can also be upregulated in pTregs in vivo, where the type of antigen-presenting cells (APCs) presenting antigen(s) to CD4^+^ T cells can trigger Helios expression [89]. Nrp-1 expression by pTregs can be induced by TGF-β during inflammation, which precludes its use to detect Tregs of thymic origin [67,90]. Thus, to date, there is no established specific marker for tTregs. Although the analysis of the markers described below, which are detected during or after thymic differentiation, helps with making assumptions about the functional state of the Treg, it is not clear whether such markers can be attributed to a separate Treg subpopulation or not.

**Figure 1:**
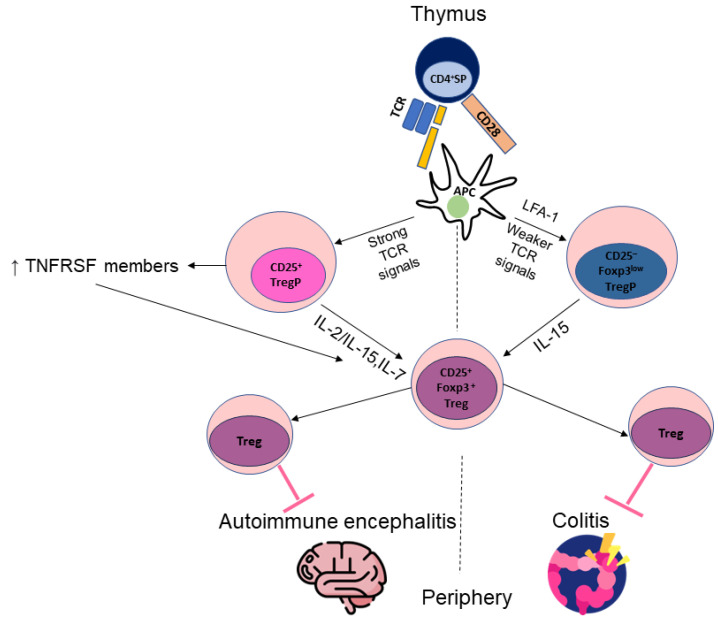
Heterogeneity in thymic development of Tregs. Two types of TregPs arise from CD4^+^ SP thymocytes. APCs present self-peptides to CD4^+^ SP thymocytes via the TCR, where intermediate levels of signaling give rise to the Treg developmental pathway. Strong signals result in negative selection. TCR- and co-stimulatory CD28-mediated signaling is required for both types of TregPs, with TCR signaling being stronger for CD25^+^ TregP cells. The development of CD25^–^Foxp3^low^ TregP cells is also dependent on LFA-1, which enhances TCR signaling. The TCR-dependent stage induces the expression of TNFRSF members by CD25^+^ TregP cells. They increase their sensitivity to IL-2. In the TCR-independent stage, CD25^+^ TregP cells convert into mature CD25^+^Foxp3^+^ Tregs. This process is dependent on IL-2 or on the related common γ-chain cytokines IL-15 and IL-7. CD25^–^Foxp3^low^ TregP cells require IL-15 at this stage. FoxP3 expression further upregulates the expression of other Treg-associated molecules. Mature Tregs derived from two types of TregPs show functional differences. Tregs from CD25^+^ TregP cells prevent experimental autoimmune encephalitis, whereas Tregs from FOXP3^low^ TregP cells are able to suppress colitis.

Two differentiation pathways of two-step thymic Treg cell development have been described [91] (Figure 1). A classical pathway proposes the development of a CD25^+^Foxp3^-^ cell population containing Treg cell precursors from self-reactive CD4+ single-positive (SP) thymocytes. Immature thymocytes carry T cell receptors (TCRs) that recognize self-peptides presented by thymic APCs. As this happens, some thymocytes are negatively selected, whereas others differentiate into Tregs. However, the factors that determine thymocyte fate are poorly understood. The axis of Treg development is reported to be based on a combination of TCR-mediated, CD28-dependent and cytokine signals. Thus, the first step is dependent on signaling through TCRs, which initially leads to the upregulation of CD25. The strength of the TCR signal is important and correlates with the subsequent expression of tumor necrosis factor receptor superfamily (TNFRSF) molecules such as GITR, OX40 (CD134) and TNFR2 (CD120b). The combined deficiency of these TNFRSF members is associated with impaired tTreg development [92]. Importantly, OX40 expression correlates with early stages of human Foxp3^+^ thymocyte differentiation. Its expression is observed at the CD4^+^CD8^+^ double positive stage and then at the CD4^+^ and CD8^+^ SP stages. OX40-mediated signaling drives the maturation of tTreg precursors and induces the proliferation of tTreg precursors and mature tTregs [93]. Constitutive expression of GITR regulates the size, composition and further maturation of Tregs [46]. TNFR2 is upregulated during stimulation; its expression remains consistently high and is critical for the activation, expansion and phenotypic stability of Tregs [52]. Moreover, TNFR2 is important for FoxP3 expression. Reduced Foxp3 expression has been demonstrated in TNFR2-deficient mice [94]. FoxP3 expression depends on the methylation status of several regions at the *FoxP3* locus [95]. TNFR2-deficient mice show increased levels of cytosine-phospho-guanine (CpG) methylation at the *FoxP3* promoter, suggesting that TNFR2 signaling supports demethylation in this region, maintaining optimal FoxP3 levels for Treg differentiation and stabilization [94]. Signaling by TNFRSF members helps make Treg progenitor cells (TregP cells) much more sensitive to cytokine interleukin 2 (IL-2) [92]. Importantly, stable and high expression of CD25 is required for the further formation of high-affinity receptors for IL-2. CD28 signaling is likely to ensure the optimal differentiation or survival of those thymocytes that have received appropriate TCR-dependent signals [88]. At the second stage, which is TCR-independent, CD25^+^ TregP cells convert into mature CD25^+^Foxp3^+^ Tregs. This process depends on IL-2 or on the related common γ-chain cytokines IL-15 and IL-7 and the transcription factor STAT5 [88]. TCR-independent tTreg maturation and proliferation in an IL-2-dependent manner are critically dependent on OX40 [93]. The induction of FoxP3 expression consequently leads to the upregulation of molecules such as CTLA-4, CD39 and CD137. CTLA-4 is constitutively expressed by Tregs [43]. CD39 expression on Tregs shows high inter-individual variation and is particularly high at sites of inflammation [40]. CD137 is highly expressed by Tregs and defines a Treg activation signature (CD137^+^CD154^-^) that allows rapid identification and sorting of epigenetically stable antigen-activated FoxP3^+^ Tregs for subsequent cell culture manipulations. The expression of LAP and GARP requires prolonged stimulation and, interestingly, does not necessarily correlate with FoxP3 expression [65]. TIGIT, which is highly expressed after Treg activation, defines the stability of this cell lineage [96]. The expression of GPA33^+^ gradually increases on cells upon maturation. During the final stages of thymic development, tTregs acquire CD31, and just before they enter the peripheral blood, the expression of GPA33 is significantly upregulated [63]. In addition, its expression remains stable.

The second pathway of thymic Treg cell development involves maturation from Treg cell progenitors with initially low expression of Foxp3 and no detectable expression of CD25 (CD25^–^Foxp3^low^ TregP cells) [91]. Activation of the transcription factor NF-κB is required for the generation of these TregP cells as well as for classical TregP cells. CD25^–^Foxp3^low^ TregP cells are characterized by the high expression of GITR and OX40 and are converted into mature CD25^+^Foxp3^+^ Tregs upon stimulation with IL-15 [97].

Interestingly, the maturation processes of the two progenitor populations have been shown to differ in many aspects. They have distinct TCR repertoires, different affinities for self-antigens in the thymus, and unique transcriptomes. In addition, the differentiation kinetics of the two populations are different, being longer for Foxp3^low^ TregP cells [91]. This is because Foxp3^low^ TregP cells are more dependent on stable interactions between T cells and APCs or on co-stimulation with adhesion molecules such as lymphocyte function-associated antigen 1 (LFA-1), which helps prolong T cell–APC contacts and enhance TCR signaling [91,98]. Importantly, mature Treg cells derived from both types of progenitors show distinct functions in suppressing autoimmunity. Treg cells derived from CD25^+^ TregP cells prevent experimental autoimmune encephalitis, whereas Tregs derived from FOXP3^low^ TregP cells are able to suppress colitis [91]. The existence of two types of Treg progenitors in the thymus perfectly reflects the heterogeneity of tTreg development. However, there is evidence that two types of Treg progenitors are required for the maintenance of immune tolerance by mature Tregs, making these subpopulations a potential target for the treatment of autoimmune diseases.

Although two types of Treg progenitors have been described, it remains unclear how the precursors of these progenitors are generated. Previously, a CD4^+^GITR^+^CD122^+^CD25^-^Foxp3^-^ cell population was reported to be the precursor of classical CD25^+^Foxp3^-^ TregP cells. The transition to CD25^+^Foxp3^-^ TregP cells is mediated by a c-REL-dependent mechanism [99]. c-REL regulates CD25 induction in CD25^+^Foxp3^-^ TregP cells and contributes to some extent to its induction in CD25^–^Foxp3^low^ TregP cells. However, it remains to be determined whether the CD4^+^GITR^+^CD122^+^CD25^-^Foxp3^-^ cell population can be the precursor of CD25^–^Foxp3^low^ TregP cells [100].

### 4.2. Peripheral Development

As mentioned above, Tregs can be developed extrathymically in the periphery. pTregs have great potential to be used for various therapeutic purposes. It is important to emphasize that the peripheral pool of Tregs consists of natural Tregs (developed in the thymus) and peripheral Tregs themselves (developed from CD4^+^CD25^−^Tconv cells). Both types of Tregs are important for maintaining immune tolerance, but the origin of differentiation can define Treg function and stability [77]. Therefore, it is crucial to highlight the peculiarities of pTreg cell development.

It is well established that pTregs can develop from Tconv cells in response to a combination of TCR-dependent non-self-antigen and cytokine stimulation, including IL-2 and TGF-β. Their generation is also promoted by factors such as retinoic acid and the alarmin IL-33 [23]. As they differentiate following the recognition of antigens from food or commensal microbiota, their abundance on mucosal surfaces increases [101]. pTregs are also developed during pregnancy to support fetal–maternal tolerance [23]. However, in-depth knowledge of pTreg generation in the context of pTreg origin does not seem to be fully clear, reflecting the lack of reliable markers for distinguishing pTregs from mixed Treg populations (consisting of peripheral and thymic Tregs) in peripheral lymphoid tissues [102]. This is because tTregs can migrate from the thymus and accumulate in tissue under inflammatory conditions. Both tTregs and pTregs share T cell surface activation markers, such as CD25, CTLA-4, GITR, LAP and GARP [103], making the separation of the phenotypic characteristics of pTregs complex. In addition, the phenotype of pTregs is dependent on the surrounding microenvironment, which, in some cases, defines the instability of this cell lineage. We can observe Treg plasticity under inflammatory or lymphopenic conditions, when pTregs can acquire the phenotype of pathogenic effector cells [102]. A study on lymphopenic mice demonstrated the lymphopenia-induced downregulation of Foxp3 expression in natural Tregs [104]. This consequently led to the loss of their suppressive function, the acquisition of IL-2 expression and a gained potential to differentiate into Th cells with the subsequent release of effector cytokines (IL-17, IL-4, TNF-α and interferon γ (IFN-γ)) [104]. Moreover, transient or unstable expression of FoxP3 has been observed in pTregs under homeostatic and autoimmune conditions with strong self-antigen engagement [105]. These “exFoxp3“ cells are CD25^−^, GITR^low^ and CD127^high^ with heterogeneous expression of folate receptor 4 (FR4), CTLA-4 and CD103. FR4 is responsible for the proliferative capacity of Tregs, as shown in mice [69]. CD103^+^ Tregs influence local immune regulation in inflamed tissues, as shown in mice [72]. TCR repertoire analyses revealed that exFoxp3 cells develop from both natural and pTregs and share an activated memory phenotype with heterogeneous CD62L expression and high CD44 expression. It was also shown that a significant number of these cells produce IFN-γ, and some cells produce IL-17A [105]. Interestingly, a small subset of ICOS^+^ Tregs has also been shown to produce IFN-γ and IL-17 in normal human peripheral blood and in mice [81]. The molecular mechanisms defining Treg instability should be fully elucidated in the future. The identification of pTreg stability and the development of approaches to improve it are therefore crucial for clinical applications.

### 4.3. More Plasticity during Differentiation

**Figure 2:**
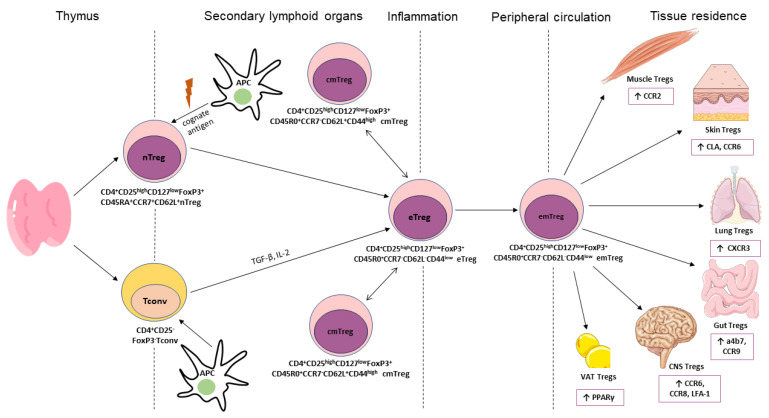
Plasticity of Tregs during differentiation. Thymic precursors give rise to nTregs and Tconv cells. In SLOs, they are activated by antigen presentation driven by APCs. Strong antigen stimulation leads to the differentiation of nTregs into eTregs, which enter the periphery. Tconvs that have received appropriate antigen stimulation also require TGF-β and IL-2 for further differentiation into Tregs. Under inflammatory conditions, they are activated and differentiate into eTregs. The differentiation of eTregs into cmTregs occurs with their retention in SLOs with the upregulation of homing molecules such as CD62L and CD44. emTregs are cells that have responded to antigens and that can survive for long periods in the absence of antigens in the peripheral circulation or in non-lymphoid tissues. Tissue homing of emTregs is associated with the expression of chemokine receptors and adhesion molecules that define the presence of specialized tissue-resident Treg subsets in the muscle, skin, lung, gut, central nervous system (CNS) and VAT.

Based on their differentiation status, Tregs can also be classified as naïve (nTregs), effector (eTregs), effector memory (emTregs) or central memory (cmTregs) [9]. nTregs have not yet encountered a cognate antigen and reside in secondary lymphoid organs (SLO). nTregs have not yet encountered a cognate antigen and reside in secondary lymphoid organs (SLO). Upon exposure to an antigen, they become activated and differentiate into eTregs. While residing in SLOs, eTregs differentiate into cmTregs. Further differentiation of eTregs into emTregs occurs with the migration of the cells into the tissue or into the peripheral circulation in response to the antigen [9]. Such processes are associated with the phenotypic plasticity of Tregs. nTregs can be characterized by the expression of CD45RA, which they acquire in the final stages of thymic development, whereas memory Tregs typically express CD45RO. In addition, both cmTregs and nTregs express the migratory marker L-selectin (CD62L), which is required for retention in SLOs. Moreover, CD44 is highly expressed by cmTregs and poorly expressed by effector Tregs. nTregs also express high levels of the chemokine receptor CCR7, whereas cmTregs and emTregs downregulate its expression [75]. Hence, nTregs can be classified as CD4^+^CD25^high^CD127^low^FoxP3^+^CD45RA^+^CCR7^+^CD62L^+^, cmTregs can be classified as CD4^+^CD25^high^CD127^low^FoxP3^+^CD45R0^+^CCR7^-^CD62L^+^CD44^high^, and eTregs with emTregs can be classified as CD4^+^CD25^high^CD127^low^FoxP3^+^CD45R0^+^CCR7^-^CD62L^-^CD44^low^ T cells (Figure 2). The phenotype of tissue Tregs is already primed within SLOs prior to terminal differentiation in tissues, where they acquire a proper tissue-specific phenotype [106]. For example, visceral adipose tissue (VAT) Tregs are characterized by the expression of the VAT Treg-specific transcription factor PPARγ [107]. Within SLOs, a small number of PPARγ-expressing Tregs emerge, but they only become PPARγ^high^ and acquire the full VAT Treg phenotype and epigenetic profile only when they enter the VAT [108,109]. The homing of Tregs in tissues is controlled by the increased expression of certain chemokine receptors and adhesion molecules [106], which is also shown in Figure 2.

In summary, elements of heterogeneity and plasticity are observed during the development of both thymic and peripheral Tregs. The manifestation of plasticity is more prevalent in pTregs, as they are more dependent on the surrounding microenvironment. The molecular mechanisms defining Treg heterogeneity, plasticity and thus instability should be fully elucidated in the future. The development of approaches to improve pTreg stability is essential for clinical applications.

## 5. Markers Associated with Suppressive Activity of Tregs

As key players in supporting immune tolerance, Tregs have the ability to suppress effector lymphocytes. Several mechanisms of Treg-mediated suppression have been identified, including the secretion of inhibitory cytokines and granzymes, the implementation of suppressive activity through intercellular contact, the disruption of effector T lymphocyte metabolism and the inhibition of telomerase [110,111,112]. Effective suppressive activity is supported by the expression of specific markers that can influence different suppressive mechanisms. These markers can either support Tregs only under certain circumstances, e.g., in inflammatory diseases, or they can be permanent, with stable expression over time.

First of all, it is important to emphasize that the master protein responsible for the acquisition of the suppressive function by Tregs is FoxP3. As mentioned above, mutations in the FoxP3 gene result in IPEX syndrome [113]. The expression of FoxP3 is discontinuous and largely depends on the methylation level of the CpG island in the genomic region of the FoxP3 locus. However, FoxP3 expression is also supported by specific markers (Table 4). In addition to FoxP3, the expression of an impressive number of other markers influences Treg-mediated suppression. TIGIT^+^ Tregs are highly suppressive and may represent a functionally distinct subpopulation [49]. This is due to the ability of TIGIT to directly suppress T cell responses independently of APCs [48]. In addition, TIGIT expression is associated with increased expression of Treg effector molecules, as shown in vitro. Compared to TIGIT^-^ Tregs, TIGIT^+^ Tregs express more granzyme B, IL-10 and fibrinogen-like protein 2 (Fgl2) [49]. Interestingly, TIGIT^+^ Tregs specifically inhibit pro-inflammatory Th1 and Th17 cells but not Th2 cell responses [49]. TNFR2 expression is continuous and critical for the suppressive function of Tregs under normal and inflammatory conditions in a mouse model [52,94]. CD39^+^ Tregs are highly suppressive, and Tregs from CD39-deficient mice demonstrate impaired suppressive activity [37]. Similarly, Gal-1-deficient Tregs show markedly compromised suppression of target cells, indicating that Gal-1 also plays an essential role in maintaining Treg-mediated tolerance [85]. The subset of FoxP3^+^ Tregs with the highest levels of CD25 also have the phenotype with the strongest suppressive function [35]. CD127 expression on Tregs is variable and depends on their localization and activation status. With low-to-absent levels of CD127, CD4^+^CD25^+^CD127^low/−^ Tregs express the highest levels of Foxp3 and have an excellent suppressive capacity [114]. A study using murine GARP-deficient Tregs demonstrated the instability of the GARP^-^ Treg phenotype [115]. The absence of GARP resulted in the downregulation of CD25, Nrp-1, CTLA-4 and IL-10 and to the impaired suppressive activity of Tregs [115]. Interestingly, the link between GARP and FoxP3 remains controversial [61]. First, a mutual dependency of GARP and FoxP3 expression was described [116], but more recent studies have shown that they are not regulated by each other [61]. LAP^+^GARP^+^ Tregs are fully suppressive, and with the application of GARP or LAP repurification, a highly purified, CpG demethylated Treg population can be obtained for potential clinical purposes [54]. CTLA-4 defines Tregs with potent suppressive activity, whereas CTLA-4-deficient Tregs show no suppressive activity [43]. CTLA-4 binds to costimulatory CD80/86 molecules on the surface of dendritic cells (DCs), preventing their interaction with CD28 on effector T cells and thereby blocking their activation. In addition, the ligation of CTLA-4 to CD80/86 induces competent DCs to express indoleamine 2,3-dioxygenase (IDO) [117]. IDO is an enzyme that catalyzes the conversion of the essential amino acid tryptophan to N-formyl-kynurenine, which is then converted into various downstream metabolites [118]. It appears that IDO acts as an immune regulator for T cells, suggesting that the IDO mechanism of CTLA-4^+^ Tregs is another contributor to appropriate suppressive activity and immune tolerance. The expression of LAG-3 may stimulate Treg-mediated suppression. Moreover, LAG-3 may synergize with CTLA-4 and PD-1 to enhance the suppressive activity of Tregs [119]. Recent studies have highlighted the importance of the ICOS-ICOSL signaling pathway for Treg-mediated self-tolerance [81]. Interestingly, GITR, on the contrary, acts as an inhibitor of the suppressive function of Tregs, as shown both in vitro and in vivo [120].

In summary, Treg-mediated suppression is maintained by a number of markers. Their absence can lead to either a significant reduction in or loss of suppressive activity. In addition to the traditional CD4^+^CD25^high^CD127^low^FoxP3^+^ phenotype used to isolate and expand Tregs, other markers can be used to improve cell purity, stability and function. These effects lead to the emergence of functionally diverse Treg subsets, a clear example of Treg plasticity.

## 6. Many Faces in Pathology

### 6.1. Tregs in Cancer

As mentioned above, Tregs are central players in the regulation of immune balance and loss of self-tolerance in autoimmune diseases, transplantation and cancer. Numerous studies have been devoted to defining specific Treg subsets and observing alterations in Treg biology in the pathogenesis of certain diseases. As cancer progresses, there is usually an upregulation of Treg numbers, which is a hallmark of a poor prognosis. Tregs can inhibit the anti-tumor cytotoxic activity of CD8^+^ T cells, NK cells and NKT cells, thereby leading to anti-tumor cell exhaustion, immune evasion and tumor progression [121]. The tumor microenvironment (TME) induces the adaptation of Tregs to new acidic, hypoxic and nutrient-poor conditions, represented by metabolic changes and mechanisms that facilitate the survival, stability and abundance of Tregs in the TME [121]. Such changes correlate with altered expression of Treg markers, leading to the emergence of novel phenotypes as a manifestation of Treg plasticity. For example, the specific expression of interferon regulatory factor 4 (IRF4) by intra-tumoral Tregs in melanoma, lung and liver cancers marks Tregs with superior suppressive activity compared to IRF4^-^ Tregs [122]. IRF4^-^ Tregs produce reduced levels of effector and suppressive molecules, such as ICOS, CTLA-4 and IL-10. Also, increased levels of markers such as TIGIT, Nrp-1, neurogenic locus notch-homolog protein 1 (Notch-1), T cell immunoglobulin and mucin domain 3 (TIM-3), B-lymphocyte-induced maturation protein 1 (BLIMP-1) and others (shown in Table 5) also contribute to enhanced Treg activity and the promotion of tumor growth. The accumulation of Tregs in tumors is chemokine-dependent and is mediated by the upregulated expression of C-C motif chemokine receptor 8 (CCR8), CCR4, CCR6 and IL-33 receptor (ST2) in multiple human cancers. ST2-expressing Tregs demonstrate a distinct phenotype with increased expression of CXCR3, CCR5 and CCR9 [123]. Intriguingly, the IL-33/ST-2 pathway has been proposed to contribute to Treg plasticity in colorectal cancer. IL-33/ST-2 signaling stabilizes the phenotype of IL-17^-^FoxP3^+^ Tregs and may promote the conversion of pathogenic IL-17^+^CD4^+^ T cell types into IL-17^-^FoxP3^+^ Tregs. This is supported by the observation of increased IL17a transcript levels in tumors of *St2−/−* mice [123]. Moreover, such a plastic balance between anti-inflammatory IL-17^-^FoxP3^+^Tregs and pro-inflammatory IL-17-producing Th17 cells is not only observed in cancer but also in autoimmunity and transplantation, where IL-17-producing T cells contribute to disease progression and graft rejection. In addition, TGF-β mediates the accumulation of T-bet^+^Th1-like Tregs in lung carcinoma, which are converted from IFN-γ-producing antitumor T-bet^+^Th1 CD4^+^ T cells [124], also serving as a manifestation of Treg plasticity.

### 6.2. Tregs in Autoimmunity

**Figure 3:**
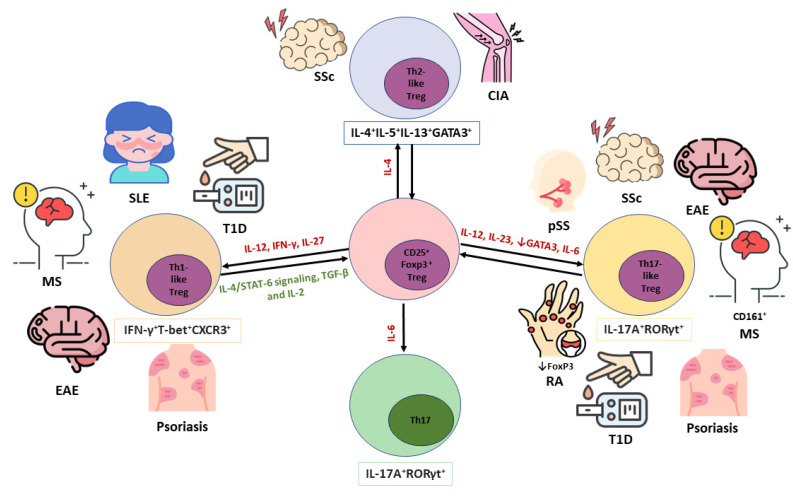
Plasticity in autoimmune diseases. Conventional Tregs have the ability to convert into pro-inflammatory Th-like Tregs and Th17 under certain factors (shown in red). Restraining factors are shown in green. Th1-like Treg phenotype is observed in T1D, SLE, MS, experimental autoimmune encephalomyelitis (EAE) and psoriasis, Th2-like in systemic sclerosis (SSc) and collagen-induced arthritis (CIA), Th17-like in pSS, SSc, EAE, MS, RA, T1D and psoriasis.

Autoimmune diseases are characterized by a loss of self-tolerance, and Tregs are essential for restoring immune balance. In diseases such as multiple sclerosis (MS) [125,126], systemic lupus erythematosus (SLE) [127], type 1 diabetes (T1D) [128], myasthenia gravis [129], rheumatoid arthritis (RA) [130], primary Sjögren’s syndrome (pSS) [131] and others [132], a decrease in the number of Tregs and/or their impaired function has been observed, leading to an immune response and disease progression. In RA, increased expression of CCR4 and CCR6 reflects the ability of Tregs to migrate into inflammatory joints and is associated with disease severity [133]. As mentioned above, Treg plasticity has been described in some autoimmune diseases, including RA, where Tregs can reversibly convert into Th-like cells. Th-like Tregs display the key characteristics of conventional Tregs, including the expression of FoxP3, but they produce pro-inflammatory molecules that are typically expressed by Th cells [22]. In RA, the IL-6/STAT3 pathway significantly promotes the conversion of Tregs into pro-inflammatory Th-like cells [134]. Such conversion results in the loss of FoxP3 expression and the ability to secrete IL-17. IL-17 promotes osteoclastogenesis in the synovial fluid of RA patients [135]. In addition, a deficiency of tumor necrosis factor receptor-associated factor 6 (TRAF6) facilitates the conversion of Tregs to IL-4^+^IL-5^+^IL-13^+^GATA3^+^ Th2 cells, which causes autoimmune arthritis in mice [136]. In MS, an imbalance of the Th17/Treg ratio correlates positively with the severity of symptoms [137]. The number of IL-17A^+^RORγt^+^Th17-like Tregs and FoxP3^low^ Tregs increases in clinically isolated syndrome, which, together with impaired Foxp3 and Helios expression in Tregs and follicular regulatory T (Tfr) cells, is an early hallmark of the disease [138]. Also, IFN-γ^+^T-bet^+^CXCR3^+^Th1-like Tregs have also been detected in MS [139]. In type 1 diabetes, the plasticity of Tregs is demonstrated by a Th1-like Treg phenotype. In SLE, suppressor of cytokine signaling 1 (SOCS1) contributes to the stability of Tregs by preventing the secretion of IL-17 and IFN-γ. In addition, signal transducer and activator of transcription 3 (STAT3) reprograms Tregs into Th17-repressing cells in SLE mice, thereby ameliorating the disease [140]. IL-2, TGF-β, IL-4/STAT-6 and TNRF2 signaling have also been described as restraining regulators of Treg plasticity that help to prevent Treg conversion to a Th-like phenotype under inflammatory conditions [22,94]. It is important to gain a more accurate view of Treg stability, which is controlled by DNA methylation mediated by a family of DNA methyltransferases (DNMTs). Treg lineage stability is supported by Treg-specific CpG hypomethylation patterns at specific genomic loci, including the *Foxp3* locus and others [141,142]. Although the epigenetic mechanisms regulating Treg stability are not fully understood, it has been shown that epigenetic regulators such as ubiquitin-like with plant homeodomain and RING finger domains 1 (Uhrf1) are required for stabilizing Treg identity [141]. Uhrf1 recruits DNMT1 to hemi-methylated DNA during DNA replication, thereby contributing to the maintenance of CpG methylation patterns [143]. The loss of Uhrf1 results in the generation of ex-Foxp3 cells that display an over-activated and Th1-skewed inflammatory profile with a distinct inflammatory transcriptional program, supporting the statement that a loss of maintenance DNA methylation causes Treg-intrinsic dysregulation of gene expression and a subsequent loss of stability [141]. These results represent an important field for further exploration and investigation of epigenetic modifiers. The balance between conventional and Th-like Tregs in autoimmunity is shown in Figure 3. Unfortunately, the mechanisms that drive the secretion of pro-inflammatory cytokines by Th-like Tregs are not known, and further studies are needed on the factors that contribute to Treg stability and the maintenance of their anti-inflammatory function.

### 6.3. Tregs in Transplantation

Allograft tolerance is one of the main challenges in the field of transplantation. Immune-mediated injury remains the major cause of late allograft rejection. In solid organ transplantation, a high Tregs/Tconvs ratio is important for maintaining tolerance [144]. Tconvs drive transplant rejection, whereas Tregs have the ability to limit their expansion [144]. Therefore, it is important to highlight the phenotypic characteristics of Tregs that influence their function in transplantation. First, there is FoxP3-mediated upregulation of CCR4 and CCL3, which modulate Treg recruitment to the allograft and their proximity to Tconvs [144]. Second, the upregulation of some other Treg markers and cytokines drives a tolerogenic milieu. For example, the expression of Notch-1 by Tregs is increased in solid organ transplantation, and the inhibition of Notch-1 enhances the survival, proliferation and suppressive function of Tregs, making Notch-1 a potential target for improving allograft tolerance [145]. Furthermore, increased expression of TGF-β and IL-10 was observed, which also promote tolerance. In addition, the adoptive transfer of Tregs could improve the condition of patients with GvHD [146], which is a common complication in hematopoietic stem cell transplantation [147]. Specifically, increased expression of Treg markers, including TIM3, PD-1, ST2, LAP and CD150, has been observed in GvHD. Their upregulation contributes to Treg-mediated suppression and, in the case of CD150, to the quiescent state of hematopoietic stem cells. This results in the inhibition of GvHD and successful engraftment [146]. The Th-17-like plasticity of Tregs, which was previously discussed in detail, is also observed here. In the presence of pro-inflammatory cytokines such as IL-1, IL-2, IL-15, IL-21 and IL-23, Tregs can upregulate the Th17 lineage transcription factor and secrete IL-17, thereby promoting acute and chronic allograft rejection [148].

The instability and plasticity of Tregs can be observed under the described conditions, which may play a dual role. Enhancing Treg function may either limit the expansion of autoreactive cells in autoimmunity and facilitate graft engraftment or promote tumor immune evasion. Therefore, the modulation of Treg plasticity should be considered in the development of therapeutic approaches. The following table presents representative markers of Tregs and their combinations to define their role in different pathologies.

**Table 5:** Markers and their combinations in cancer, autoimmunity and transplantation.

Marker or Combination	Disease	Association	Reference
Granzyme B (GZMB)	Colorectal cancer	Elevated expression, mediates tumor immunity.	[149]
TIM-3	Colon carcinoma, melanoma	Marks intra-tumoral Tregs. TIM-3^+^ Tregs have enhanced suppressive function and promote neoplastic growth.	[150,151]
	Rheumatoid arthritis	Reduced TIM-3 expression is associated with decreased functional activity.	[152]
	GvHD	Elevated TIM-3 expression is associated with increased functional activity.	[153]
TIM-3 and PD-1	Lung cancer, melanoma and myeloid leukaemia	Enhanced expression in tumors. Combination of anti-TIM-3 therapy with anti-PD-1 therapy can be effective in eliminating resistance to chemotherapy or potentiating each other.	[154,155]
PD-1	Melanoma, hepatocellular carcinoma, non-small cell lung cancer (NSCLC), colorectal cancer, gastric and liver cancer	Elevated expression in tumors. The role of PD-1/PD-L1 signaling for Tregs is not fully clear.	[155]
	Systemic lupus erythematosus	High expression of PD-1 on Tfrs with dysfunction of suppressing Tfhs proliferation and activation.	[156]
	Chronic GvHD	Increased PD-1 expression follows Treg proliferation in patients receiving low-dose IL-2.	[157]
LAG-3 and TIM-3	Colorectal cancer	Higher expression of TGF-β, IL-10 and CTLA-4 by LAG-3 + TIM-3 Tregs.	[158]
CCR8	Breast cancer, colon cancer, lung cancer, melanoma and angiosarcoma	Upregulated by tumor-resident Tregs. High expression of CCR8^+^ Tregs is associated with poor prognosis. Targeting CCR8^+^ Tregs inhibit tumor growth.	[159]
IRF4	Melanoma, lung cancer and liver cancer	Identifies effector intra-tumoral Tregs with enhanced suppressive potential capable of promoting tumor growth in vivo. Abundance of IRF4^+^ Tregs correlate with poor prognosis.	[122]
CCR8 and ICOS and IRF4	Lung cancer	Elevated expression of IRF4 contributes to enhanced Treg suppressive activity and worse prognosis in non-small-cell lung cancer.	[122]
BLIMP-1	Melanoma	Elevated expression in tumors correlates with increased suppressive function of Tregs and higher levels of Foxp3, Helios and CTLA-4. Deletion of Blimp-1 in Treg cells remodels the tumor microenvironment and sensitizes the tumors to anti-PD-1 treatment.	[160]
TNFR2	Colorectal, lung, gastric, cervical and ovarian cancer; acute myeloid leukemia and melanoma	Elevated expression contributes to enhanced Treg suppressive activity and worse prognosis.	[161]
	Rheumatoid arthritis	TNFR2 is upregulated in rheumatoid arthritis fibroblast-like synoviocytes (FLS) and promotes inflammatory chemokine and cytokine expression in FLS.	[162]
CD30 and OX40 (CD134)	Colorectal cancer	Elevated CD30 and OX40 expression. Colorectal cancer-infiltrating CD30^+^OX40^+^ Treg subset could be an indicator of patient survival and may be used as a potential biomarker to diagnose and prognosticate colorectal cancer.	[163]
GARP	Lung cancer, colon cancer, gastric cancer and melanoma	Elevated expression on Tregs. Infiltration of Tregs expressing GARP in the tumor microenvironment is associated with poor prognosis. Infiltrating Tregs from early-stage patients with lung cancer exhibit higher GARP expression than those from advanced cancer patients, which indicates that GARP might be an early prognostic biomarker.	[164,165]
Forkhead box protein A1 (FOXA1)	Lung cancer	High expression of FOXA1 but low levels of FOXP3. FOXA1^+^ Tregs inhibit the antitumor immunity of T cells.	[166]
TNFRSF9	Lung cancer	Low expression is associated with an enhanced survival rate.	[167]
Layilin (LAYN)	Liver cancer	Increased expression of LAYN, TNFRSF9 and ICOS. Small numbers of LAYN^+^ Tregs.	[168]
LAYN and FoxP3 and Helios	Liver cancer	Upregulation of LAYN expression in FoxP3^+^Helios^+^ Tregs.	[169]
GITR	Glioblastoma, lung cancer and bladder cancer	Elevated expression. Targeting GITR in combined therapy seems to be a promising approach.	[45,170]
ICOS	Liver cancer, melanoma, head and neck squamous cell cancers, gastric cancers, breast cancer, ovarian cancer, clear cell renal cell carcinoma and acute myeloid leukemia	Elevated expression correlates with bad outcomes in most cases.	[81]
	Type 1 diabetes	Decreased expression. ICOS^+^ Tregs involved in prevention of diabetes.	[81]
	Inflammatory bowel disease	Marks Treg subset located in intestinal tissue, which is more suppressive and indicative of the positive outcome of IBD.	[81]
	Rheumatoid arthritis	Elevated expression. Suggested to be used as a predictor of responses to treatment for methotrexate non-responsive patients.	[171]
	Sarcoidosis	High expression levels of ICOS in lung Tregs of pulmonary sarcoidosis patients and in patients with Lofgren’s syndrome, thus associating the degree of ICOS expression on Tregs with prognosis of sarcoidosis.	[172]
CCR6	Liver, colorectal and renal cancer	High expression is correlated with Treg migration into tumor tissue and poor prognosis.	[173]
CCR4	Breast and prostate cancer	Elevated expression by tumor-infiltrating Tregs. Overexpression of FoxP3 increases CCR4^+^ Treg infiltration in breast cancer, resulting in a decreased anti-tumor immune response.	[174,175]
Vascular endothelial growth factor receptor 2 (VEGFR2)	Gastric cancer	High expression by eTregs and higher Ki67 expression compared with VEGFR2^−^ eTregs.	[176]
OX40 (CD134)	Mantle cell and follicular lymphomas, breast cancer, neck and cutaneous squamous cell carcinomas	Elevated expression of OX-40 and CTLA-4 on the surface of tumor-specific Tregs.	[146,177]
TIGIT	Neck squamous cell carcinoma, melanoma, bladder cancer, follicular lymphoma	Elevated expression. In head and neck squamous cell carcinoma and follicular lymphoma, it is correlated with an increased ability of TIGIT^+^ Tregs to suppress the proliferation of CD8^+^ T cells. In bladder cancer, TIGIT^+^ Tregs highly expresses IL-32 and promotes the migration and invasion of tumor cells.	[178,179,180,181]
Nrp-1	Melanoma, squamous cell carcinoma of the head and neck, cervical cancer, chronic lymphocytic leukemia	Elevated expression, being essential for maintaining intra-tumoral Tregs’ function and phenotype. Elevated levels of FoxP3 and GITR in Nrp-1^+^ Tregs. High percentage of Nrp-1^+^ Tregs is associated with poor prognosis.	[182,183]
Notch-1	Organ transplantation	Elevated expression. Notch-1 blockade results in an increase in survival, proliferation and function of Tregs, which are important for the improvement of long-term graft survival.	[145]
CXCR3	Draining lymph nodes and tumors	Elevated expression. Loss of CXCR3 boosts tumor CD8^+^ T cells and slows cancer progression.	[184]
ST2	Colorectal cancer	Elevated expression of ST2 promotes Treg accumulation in the tumor environment.	[123]
	GvHD	IL-33-mediated expansion of ST2^+^FoxP3^+^ Tregs can protect against acute graft-versus-host disease.	[185]
LAP	GvHD	Treg expansion based on selective surface expression of LAP and further adoptive transfer can delay the development of xenogeneic GVHD.	[54]
CD150	GvHD	High expression. CD150^high^ bone marrow Tregs maintain hematopoietic stem cell quiescence, pool size, and engraftment, which are associated with elevated expression of CD39, CD73 and adenosine.	[186]

It can be observed that a single marker can be present in a variety of diseases, which allows us to assume that a disease-specific Treg marker and consequently a disease-specific Treg subset do not exist. Furthermore, combinations of Treg markers are being studied to define their role in certain diseases, whereas the use of only one marker may not be representative enough. The question of whether a combination of markers can be disease-specific is not yet clear, as studies on Treg markers have certain limitations. First, each study is dedicated to one marker or a specific combination and may not take into account all existing markers and their interactions. Second, the selective choice of a marker or a combination is associated with the complexity of the process and the impossibility of considering all existing markers in one study. Third, individual variation has a significant impact on the conclusions about the definitive role of a marker or combination. The last limitation can be addressed by the use of animal standardization. Nevertheless, these limitations require further investigation and cannot be resolved at this time. It is clear that the plasticity of Tregs, resulting from the expression of multiple markers, requires targeted correction to increase stability in the development of Treg-based therapies.

## 7. Treg-Associated Cell Markers Used for Therapeutic Approaches

Given the central role of Tregs in maintaining immune homeostasis, there is a growing interest in developing therapeutic approaches that harness the potential of Tregs. In the context of transplantation and various autoimmune diseases, there is a focus on testing different approaches, including IL-2-based, polyclonal, antigen-specific and cell-engineered (CAR and TCR-transduced) approaches [28,187,188]. However, there are two major obstacles to overcome. One is a reduced number of Tregs, and the other is reduced suppressive activity of these cells. The selection of the most appropriate Treg subset for therapeutic intervention represents a further challenge. This is associated with the need to enhance the stability of a specific subpopulation for a long period of time, and with the search for a subpopulation that is most representative of the given pathological conditions, e.g., in the context of the TME. In general, it is necessary to work extensively with the stability of specific populations, modulating it in the desired direction. In this context, the concept of plasticity becomes particularly relevant. Its modulation is achieved by influencing the expression of markers. A number of approaches have been explored to induce the expression of specific markers to increase the number and suppressive activity of Tregs. In contrast, in the context of cancer, the number and suppressive activity of Tregs must be reduced. To achieve this, some Treg markers need to be inhibited. In our review, we focus on Treg-associated cell marker approaches that result in the targeted induction or inhibition of a specific Treg marker (Table 6).

**Figure 4:**
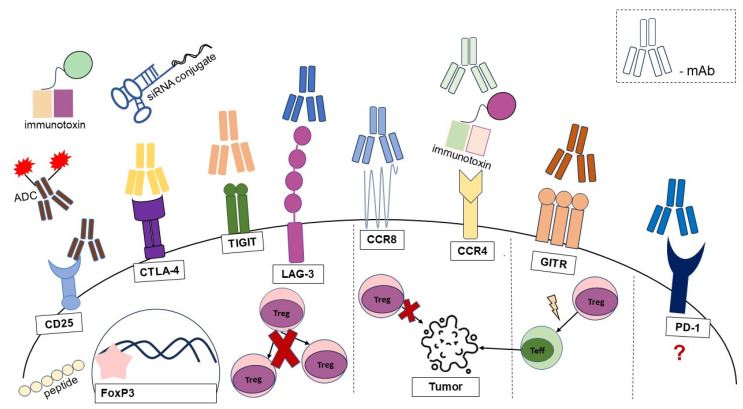
Targeting Tregs in cancer. Blockades of CD25, CTLA-4, TIGIT and LAG-3 with mAbs, siRNA conjugates (anti-CTLA-4), ADCs and immunotoxins (anti-CD25) or cell-penetrating peptides (anti-FoxP3) lead to Treg depletion. Blockades of CCR8 and CCR4 with mAbs and immunotoxins result in inhibition of Treg recruitment to the TME and enhancement of anti-tumor responses. Inhibition of GITR with mAb promotes the differentiation of Tregs into Teffs and alleviates Treg-mediated suppression of the anti-tumor immune response. The effects of the PD-1 blockade in cancer are controversial.

Monoclonal antibody (mAb)-based immunotherapy is now considered one of the most important components of cancer therapy [189]. They can directly target tumor-infiltrating cells, thereby enhancing the anti-tumor immune response. Targeting CD25 with mAb results in the depletion of Tregs. This is thought to facilitate the activation of Teff cells and thereby inhibit tumor growth, as demonstrated in breast cancer patients [190]. Another study on melanoma patients showed that Teff cells are inhibited along with Tregs, resulting in a lack of an antitumor response [191]. These findings demonstrate the limited efficacy of anti-CD25 mAb administration in cancer patients. An alternative approach is the use of antibody drug conjugates (ADCs) to selectively target CD25. ADCT-301 has been reported to induce Treg depletion and enhance antitumor immunity [192]. Its efficacy in Hodgkin’s and non-Hodgkin’s lymphomas [193,194], acute myeloid and lymphoblastic leukemia [195] and in solid tumors [196] is currently being evaluated in clinical trials. In addition, recombinant immunotoxins are being developed that target CD4, CD25 and FoxP3 in humans and that cause a reduction in Treg numbers [192]. Administration of anti-CTLA-4 mAb results in depletion of Tregs in the TME through the mechanism of antibody-dependent cellular cytotoxicity, thereby increasing the Teff/Treg ratio. Administration of agonistic anti-GITR mAb in murine glioblastoma was observed to promote the differentiation of Tregs into CD4 Teffs cells, alleviate the Treg-mediated suppression of the anti-tumor immune response and induce the generation of potent anti-tumor effector cells [45]. Interestingly, small interfering RNA (siRNA) conjugates have also been developed for the targeted inhibition of CTLA-4 on Tregs in the TME [192]. As ICOS is expressed at high levels by Tregs in cancer, clinical trials are underway to elucidate the efficacy of agonistic anti-ICOS mAbs [197]. OX40 may also be a target for Treg depletion, but it is also expressed by Teffs. In addition, OX40 agonists are used to stimulate the anti-tumor responses of Teff cells [191]. CCR4 and CCR8 antagonists inhibit the recruitment of Tregs in the TME and induce antitumor responses [198]. The role of the PD-1 blockade on Tregs remains uncertain. In several studies, a reduction in Treg-mediated suppression was observed following anti-PD-1 mAb administration. However, in some cancer patients, PD-1 inhibition results in enhanced Treg function [191]. The combination of different therapeutic approaches leads to greater efficacy of the antitumor response. Hybrid spherical nucleotide nanoparticles loaded with a CTLA-4-siRNA aptamer and PD-1 siRNA are designated to inhibit PD-1 and CTLA-4 [199]. Their administration has been shown to regulate Treg-mediated suppression and allow Teff cells to expand [199]. An aptamer chimera was generated by linking an aptamer that binds to CD137 (4-1BB) to a small antisense RNA (sasRNA) targeting FoxP3. This chimera was shown to promote an antitumor response through aptamer-sasRNA-mediated transcriptional gene silencing of FoxP3 [200]. In addition, targeting the co-inhibitory receptors TIGIT, LAG-3 and TIM-3 is also being explored as an immunotherapeutic approach. Anti-TIGIT treatment has been shown to decrease or deplete Tregs and reduce their suppressive activity, thereby contributing to anti-tumor immunity [201,202]. Administration of anti-LAG-3 mAb has also been shown to reduce the number of Tregs [203]. Figure 4 illustrates the effects of blocking Treg receptors in cancer.

The role of Treg plasticity in targeted cancer immunotherapy is ambiguous. On the one hand, targeting depends on the stability of a given marker, with a stable marker allowing for longer-term and more successful targeting. On the other hand, TME-mediated phenotypic switching of Tregs allows the targeting of “freshly” generated tumor-infiltrating subpopulations of Tregs. Therefore, it is essential to define the phenotypic changes in Tregs driven by the TME and to select the most appropriate marker or combination of markers for immunotherapy in each specific cancer.

**Figure 5:**
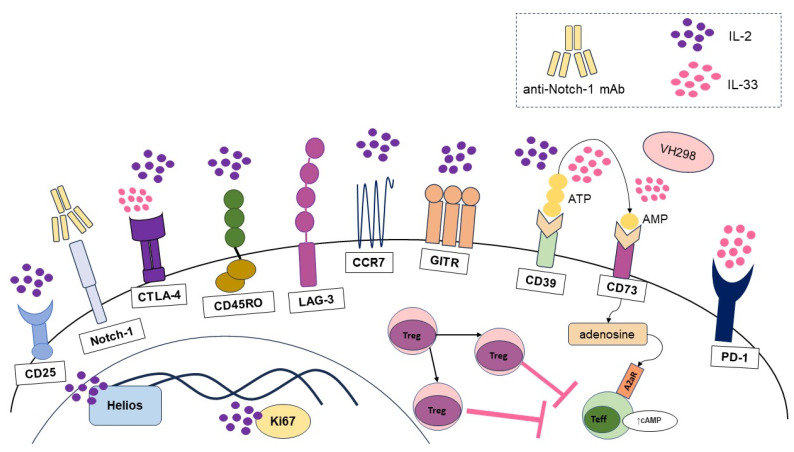
Targeting Tregs in autoimmunity and transplantation. Low-dose IL-2 stimulation of expanded Tregs results in upregulation of CD25, GITR, CTLA-4, Ki67, Helios, CD39, CD45R0 and CCR7, making Tregs more suppressive. IL-33 treatment in transplantation results in a tolerogenic phenotype of Tregs with increased expression of CTLA-4, PD-1, CD39 and CD73. CD39 and CD73 generate adenosine, which binds to the adenosine receptor A2 on the surface of Tregs, increasing the intracellular concentration of cAMP in them and leading to impaired proliferation. Inhibition of Notch-1 with anti-Notch-1 mAbs is associated with increased expression of LAG-3, CTLA-4 and Ki67 and increased TGF-β production.

In several autoimmune and inflammatory diseases, preclinical studies have demonstrated the efficacy of Treg-based cell therapy in ameliorating inflammation [204]. A number of clinical trials are currently underway to evaluate the safety and efficacy of Treg-based therapeutic approaches. Different expansion protocols exist, with IL-2-based stimulation being a commonly used approach. IL-2 plays a critical role in the development and expansion of Tregs [205,206]. Their functional activity is lost upon the removal of IL-2 signaling [207]. For instance, the responsiveness of Tregs to IL-2 in T1D is diminished, which is associated with a reduction in Treg frequency, a loss of FoxP3 expression and impaired suppressive function [208]. In vivo expanded Tregs in diseases such as T1D, SLE and ALS treated with low-dose IL-2 stimulation display upregulation of CD25, GITR, CTLA-4, Ki67, Helios, CD39, CD45R0, CCR7 and a memory phenotype, accompanied by enhanced activation and a subsequent increase in suppressive function [207] (Figure 5). Clinical studies on the effect of low-dose IL-2 in pSS, psoriatic arthritis, RA, autoimmune hepatitis, ankylosing spondylitis, Behcet’s disease, granulomatosis with polyangiitis, Takayasu’s disease, ulcerative colitis and sclerosing cholangitis have shown an increase in Treg numbers and, in most cases, clinical improvement [207,209]. Importantly, responses to IL-2 therapy vary among patients, with differences in disease stage and duration, and may also be influenced by recipient age, thymic Treg output status and peripheral differentiation status [207]. These factors highlight the need to monitor dose–response rates during treatment. Although it has been shown that IL-2 stimulation in autoimmune and inflammatory diseases results in a phenotypic shift toward a highly suppressive and therefore anti-inflammatory phenotype, further studies are needed to evaluate the influence of dose and treatment regimen on the phenotype of expanded Tregs in vivo.

Modulation of the Treg phenotype in transplantation represents another target for therapeutic intervention (Figure 5). As mentioned above, inhibition of Notch-1 may improve allograft tolerance. Indeed, mice treated with an anti-Notch-1 antibody (aNotch-1) show an increased number of Tregs in the spleen and in the graft. In addition, splenic Tregs show increased expression of LAG3, CTLA-4 and Ki67 and increased TGF-β production, resulting in enhanced suppressive activity in vitro [145]. The impact of IL-33 on Treg biology in transplantation remains uncertain. In the GvHD model, IL-33 stimulation supports the differentiation and expansion of Th1 cells while inhibiting IL-10 and Foxp3 expression, leading to lethal GvHD [210]. Another study reported that IL-33 treatment prolongs graft survival and induces the expansion of Tregs in the graft and spleen in a murine cardiac allograft model [211]. In mice receiving a skin allograft, treatment with IL-33 results in the acquisition of a tolerogenic phenotype by Tregs, expressing elevated levels of CTLA-4, PD-1, CD39 and CD73. This phenotypic switch also leads to increased expansion of Tregs in the spleen, lymph nodes and periphery, which improves long-term graft survival [212]. These observations suggest the need for further studies on the role of IL-33 in Treg-mediated tolerance. Induction therapies such as basiliximab are important in preventing acute rejection soon after transplantation [144]. Basiliximab is a non-depleting IL-2 receptor antagonist that is designed to inhibit CD25 on Teffs that mediate immune graft damage and rejection [213]. However, it also inhibits CD25 on Tregs, reduces FoxP3 and IL-10 expression and leads to reduced Treg numbers and function, therefore impairing the protective role of Tregs in preventing acute graft rejection [213]. In contrast, treatment with another induction agent rabbit anti-thymocyte globulin (rATG) allows an expansion of functionally active Tregs in vitro [214]. Moreover, a comparison of two induction therapies revealed that rATG-treated patients exhibit rATG-promoted expansion of peripheral Tregs, which likely contribute to the faster kinetics of Treg reconstitution and new thymic emigration of Tregs compared to basiliximab-treated patients [215,216]. These data may help optimize the efficacy of treatment regimens in transplantation. Another approach to enhance the suppressive function of Tregs is to upregulate CD73 expression. Increased CD73 expression by expanded Tregs may be related to their altered metabolic state and may be driven by hypoxia-inducible factor 1-alpha (HIF1A) [217]. Under resting/aerobic conditions, HIF1A is continuously degraded by the von Hippe–Lindau (VHL)-mediated ubiquitin protease pathway [217]. Modification of Treg expansion protocols with HIF1A stabilization, achieved by the use of VHL blockers, allows upregulation of CD73 expression and enhancement of Treg-mediated suppression.

**Table 6:** Targeting Treg markers for therapeutic purposes.

Marker	Treatment	Results	Reference
**Cancer**			
CD25	Anti-CD25 mAb, ADCT-301; immunotoxins 2E4-PE38, Denileukin Diftitox and LMB-2	Inhibition of CD25. Treg depletion.	[190]
CTLA-4	Anti-CTLA-4 mAb, siRNA conjugates (CTLA4apt–STAT3 siRNA, NPsiCTLA-4, cSNPs and hybrid SNPs)	Inhibition of CTLA-4. Treg depletion.	[192]
FoxP3	Immunotoxins, cell-penetrating peptides (P60, CM1315 and FOXP3 393–403), short RNA strands	Inhibition of FoxP3.	[192]
PD-1	Anti-PD-1 mAb	Controversial.	[191]
CCR4	Anti-CCR4 mAb, CCR4 antagonists, immunotoxins	Inhibition of CCR4. Inhibition of the recruitment of Tregs in the TME and enhanced anti-tumor responses.	[198]
CCR8	Anti-CCR8 mAb	Inhibition of CCR8. Inhibition of the recruitment of Tregs in the TME and enhanced anti-tumor responses.	[198]
GITR	Agonistic anti-GITR mAb	Promotion of Treg differentiation into CD4 Teffs, alleviation of Treg-mediated suppression of anti-tumor immune response, and induction of potent anti-tumor effector cells in glioblastomas.	[45]
TIGIT	Anti-TIGIT mAb	Inhibition of TIGIT results in a decrease in or depletion of intra-tumoral Tregs and in a decrease in their suppressive function.	[201,202]
LAG-3	Anti-LAG-3 mAb	Blockade of LAG-3 leads to reduction in Tregs.	[203]
**Autoimmune and inflammatory diseases**			
CD25	Low-dose IL-2	Elevated expression in in vivo expanded Tregs. Tregs have a higher suppressive function.	[207]
GITR			
CTLA-4			
Ki67			
Helios			
CD39			
CD45R0			
CCR7			
**Transplantation**			
PD-1	Exogenous IL-33	Elevated expression. Enhanced expansion of Tregs.	[212]
CTLA-4			
CD39			
CD73			
Notch-1	aNotch-1	Inhibition of Notch-1 leads to elevated expression of CTLA-4, LAG-3, Ki67 and increased TGF-β production. Increased numbers of Tregs.	[145]
CD25	Basiliximab	Blockade of CD25, reduced expression of FoxP3 leads to decreased proliferation and function of Tregs.	[213]
FoxP3			
CD73	High-dose IL-2, VLH inhibition (VH298)	Elevated expression of CD73 leads to increased Treg-mediated suppression	[217]

In summary, different Treg markers are used for targeted induction or inhibition depending on the disease. There is no universal marker that would allow the development of a perfect Treg-based treatment approach. However, it is likely that combined therapy targeting multiple Treg markers will be most effective. In cancer immunotherapy, the depletion of Tregs increases the anti-tumor response. Therefore, it is necessary to target constitutively expressed Treg markers to achieve maximal Treg depletion. In autoimmune and inflammatory diseases, as well as in transplantation, treatment strategies should aim to upregulate or induce the expression of such Treg markers that render cells more suppressive and proliferative. Although such strategies are likely to result in transient outcomes due to Treg plasticity, they lead to significant clinical improvements. Further studies are needed to improve the stability of the therapeutically induced Treg phenotype.

## 8. Conclusions

In conclusion, both heterogeneity and plasticity are observed in Tregs. In our review, we collect and analyze existing data on Treg markers and subsets in order to distinguish between two terms in normal and pathological conditions. However, it is important to note that such a distinction cannot be considered absolute, as further study of the biology of Treg markers under different conditions and their interactions is needed. It is likely that, in the future, computational methods will be integrated to help consider and reconcile information about Treg markers and subpopulations under different conditions in the context of multiple factors. This may help develop effective Treg cell products for different treatment purposes.

## Figures and Tables

**Table 4:** Markers associated with higher expression of FoxP3.

Marker	Association with FoxP3	Reference
CD39	High expression of CD39 sustains higher expression of FoxP3.	[39]
CD103	Presumably enhances TGF-β-dependent expression of FoxP3.	[72]
CD44	Costimulation with HMW-HA promotes expression of FoxP3.	[73]
PD-1	Prompts expression of FoxP3.	[87]
TNRF2	Maintains demethylation of *FoxP3* promoter.	[94]
